# Combined expression of miR-34a and Smac mediated by oncolytic vaccinia virus synergistically promote anti-tumor effects in Multiple Myeloma

**DOI:** 10.1038/srep32174

**Published:** 2016-08-24

**Authors:** Wen Lei, Shibing Wang, Chunmei Yang, Xianbo Huang, Zhenzhen Chen, Wei He, Jianping Shen, Xinyuan Liu, Wenbin Qian

**Affiliations:** 1 Institute of Hematology, the First Affiliated Hospital, College of Medicine, Zhejiang University, Hangzhou, 310003, P.R. China; 2 Clinical Research Institute, Zhejiang Provincial People’s Hospital, Hangzhou, 310014, P.R. China; 3 Department of Hematology, the First Affiliated Hospital of Zhejiang Chinese Medicine University, Hangzhou, 310006, P.R. China; 4 Institute of Biochemistry and Cell Biology, Shanghai Institutes for Biological Sciences, Chinese Academy of Sciences, Shanghai, 200031, P.R. China

## Abstract

Despite great progress made in the treatment of multiple myeloma (MM), it is still incurable. Promising phase II clinical results have been reported recently for oncolytic vaccinia virus (OVV) clinic therapeutics. One reason for this has focused on the critical therapeutic importance of the immune response raised by these viruses. However, few studies have performed their applications as an optimal delivery system for therapeutic gene, especially miRNA in MM. In this study, we constructed two novel OVVs (TK deletion) that express anti-tumor genes, miR-34a and Smac, respectively, in MM cell lines and xenograft model. The results demonstrated that the novel OVV can effectively infect MM cell lines, and forcefully enhance the exogenous gene (miR-34a or Smac) expression. Furthermore, utilization of VV-miR-34a combined with VV-Smac synergistically inhibited tumor growth and induced apoptosis *in vitro* and *in vivo*. The underlying mechanism is proposed that blocking of Bcl-2 by VV-miR-34a increases the release of cytochrome c from mitochondria and then synergistically amplifies the antitumor effects of Smac-induced cell apoptosis. Our study is the first to utilize OVV as the vector for miR-34a or Smac expression to treat MM, and lays the groundwork for future clinical therapy for MM.

Multiple myeloma (MM) is a genetically complex hematologic malignancy characterized by the accumulation of clonal malignant plasma in bone marrow. Despite remarkable progress made in the pathobiology and management of the disease, it is still incurable^1^,^2^. Deregulated expression of microRNAs (miRNAs), small noncoding RNAs that regulate gene expression, plays a key role in the pathogenesis and progression of several human cancers including MM^3^,^4^,^5^. miRNAs may act as oncogenes or tumor-suppressors. Loss of a tumor-suppressive miRNA activates oncogenic pathways that promote tumorigenic potential and drug resistance^6^,^7^. Therefore, replacing tumor suppressor miRNAs may be a potential strategy to eradicate cancer cells. In this light, recent studies have demonstrated that a variety of miRNAs such as miR-214, miR-29b, miR-125b, and miR-34a replacement strategies are active in reducing growth of MM cells^8^,^9^,^10^,^11^.

miR-34a is the first identified tumor-suppressor miRNA^12^,^13^,^14^. Indeed, miR-34a inhibits many oncogenic processes by regulating genes that function in various cellular pathways including cell cycle, p53, and Wnt signaling^14^,^15^. miR-34a has also been associated with regulation of cancer stem cell function^14^,^15^,^16^,^17^. Overexpression of miR-34a upon treatment with demethylating agent 5-Aza-2′- deoxycytidine inhibits prostate cancer stem cell growth and metastasis by directly targeting CD44^17^,^18^.

miR-34a is of special interest for myeloma miRNA therapeutics because the miR-34a promoter is hypermethylated, an epigenetic modification that typically silences gene expression, in MM patient cells and MM cell lines^19^. Further research from Wang showed that epigenetic silencing of miR-34a is caused by an lncRNA (Lnc34a), which recruits proteins that modify the gene and switches off the production of miR-34a^20^. Recent studies have demonstrated that replacement of miR-34a exerts anti-tumor activity *in vitro* and *in vivo* against human MM xenografts in animal models, which suggests an important potential clinical application^11^,^21^,^22^. However, optimization of the delivery of miR-34a is needed before it can be translated into the clinic setting.

Another factor implicated in the pathogenesis and progression of MM is Smac. Smac is a mitochondrial protein that is released into the cytosol to induce apoptosis^23^,^24^. Prior studies have established that Smac release is critical for anti-myeloma agent-induced apoptosis, and that dysfunctional Smac release contributes to cancer cell drug-resistance^24^,^25^,^26^. Recent report reveals that Smac is able to potently induce appotosis in MM by decreasing the expression of inhibitor of apoptosis (IAP) proteins^27^. Thus, regulation of Smac expression could be a promising approach to treat MM.

Oncolytic vaccinia viruses (OVVs) recently emerge as a promising novel approach to treat cancer because of their potential to infect, replicate in, and lyse tumor cells. OVVs also disseminate through the bloodstream to induce an anti-tumor immune response^28^,^29^. In fact, JX-594, an OVV with a human granulocyte-macrophage colony-stimulating factor (hGM-CSF) insertion, has moved to clinical trials, where it has shown potential in liver carcinomas and colorectal cancer^28^,^30^. In addition, recent reports have shown that an apoptin-producing recombinant VV selectively kills human cancer cells *in vitro* and *in vivo*^31^, and that VV expressing p53 effectively infects glioma cells and induces apoptosis^32^, suggesting that the recombinant “armed” OVV can be designed to express additional therapeutic genes that enhance the efficiency of destruction of the cancer cells.

In this study, we constructed OVV expressing miR-34a (VV-miR-34a) or Smac (VV-Smac), and evaluated their anti-myeloma potential as monotherapies and in combination. We found that these OVVs were able to replicate in and kill human MM cells *in vitro* and *in vivo*. Furthermore, combination therapy with VV-miR-34a and VV-Smac synergistically improved anti-tumoral efficacy.

## Results

### Construction and characterization of the novel OVVs: VV-miR-34a and VV-Smac

**Figure 1:**
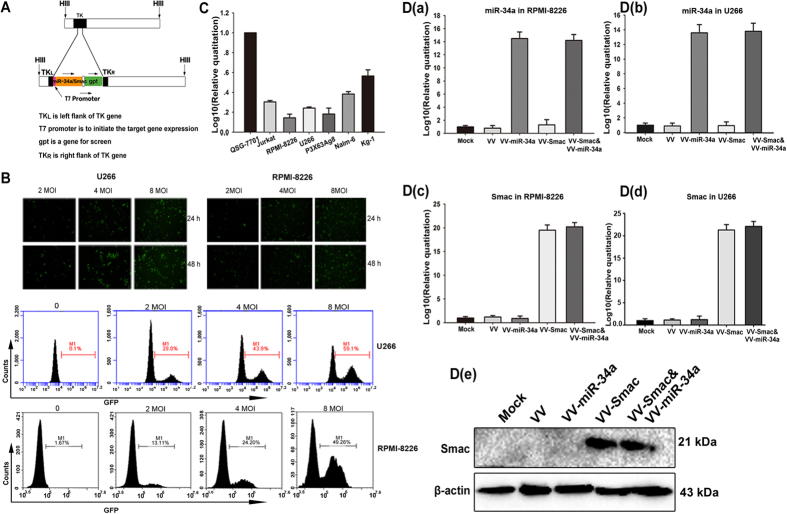
Characterization of the novel OVVs: VV-miR-34a and VV-Smac. (A) Schematic diagram of recombinant OVV structure. All viruses were constructed through homologous recombination of pCB-transgene with wild type VV in HEK293 cells. T7 promoter and gpt gene work as promoter and screen gene. (**B**) Infectious efficiency of OVV in MM cells was evaluated by fluorescence microscopy and FACS analysis. RPMI-8226 and U266 cells were infected with VV-GFP at multiple doses and time points and then observed under a fluorescence microscope (200×) (top panel). Two cell lines were treated with VV-GFP for 48 hours in dose-depended manner and then collected for FACS analysis (bottom panel). (**C**) Basal expression of miR-34a in hematologic malignancy cell lines was assessed by RT-PCR analysis (data are presented as means ± SD, n = 3). Human normal liver cell line QSG-7701 was used as the control to normalize the results. D. RT-PCR and immunoblot were carried out to analysis the enforced expression of miR-34a and Smac by OVVs. MM cell lines RPMI-8226 and U266 were infected with the indicated OVVs at a MOI of 4 for 24 hours or 48 hours. a,b. Expression of miR-34a in RPMI-8226 and U266 cells. c,d. Expression of Smac on mRNA in RPMI-8226 and U266 cells. e. Expression of Smac on protein level in RPMI-8226 cells. Full length blots were shown in Fig. S1.

The attenuated (thymidine kinase [TK]-negative) replication competent OVV vector (Western Reverve strain) was used to generate OVV expressing miR-34a or Smac (Fig. 1A). The miR-34a or Smac gene was inserted into the TK region, disrupting the function of TK. Deletion of the TK gene inhibits viral replication in normal, non-dividing cells^33^. However, cancer cells have a high concentration of functional nucleotides that enables OVV replication to occur in the absence of viral thymidine kinase. Therefore, disruption of TK results in selective replication of the OVV in tumor cells. The T7 promoter was inserted before the exogenous genes to initiate their expression, and the gpt gene works as a screen gene engineered behind the exogenous genes. The whole expression cassette was constructed into the pCB vector, which is a shuttle plasmid for VV packaging kindly provided by academician Xinyuan Liu.

To evaluate the infectious efficiency of the novel OVV on MM cell lines, U266 and RPMI-8226 cell lines were infected with VV-GFP, an OVV carrying GFP instead of miR-34a or Smac, at multiple doses and time points, respectively, and then photographed under inverted fluorescence microscopy (Nikon E300). GFP was readily detectable at 24 hours and peaked at 48 hours (Fig. 1B; upper panel). FACS analysis showed that the proportion of GFP + cells increased in a dose-dependent manner. The infectious efficiency increased to 59.1% in U266 and 49.26% in RPMI-8226, respectively, at 48 hours after infection with VV-GFP with a multiplicity of infection (MOI) of 8 (Fig. 1B; lower panel).

To verify expression of the miR-34a and Smac transegenes following OVVs infection, we examined their ectopic expression in hematologic malignancy cells using real time PCR (RT-PCR) and immunoblot. Compared to normal human liver cells QSG-7701, basal levels of miR-34a were relatively low in hematologic cell lines including MM and leukemia cells (Fig. 1C). However, infection with VV-miR-34a and VV-Smac increased the expression of miR-34a and Smac in MM cell lines U266 and RPMI-8226 remarkably on an mRNA and protein level (Fig. 1D).

### VV-miR-34a and VV-Smac synergistically induce apoptosis through activation of the caspase pathway in MM cells

**Figure 2:**
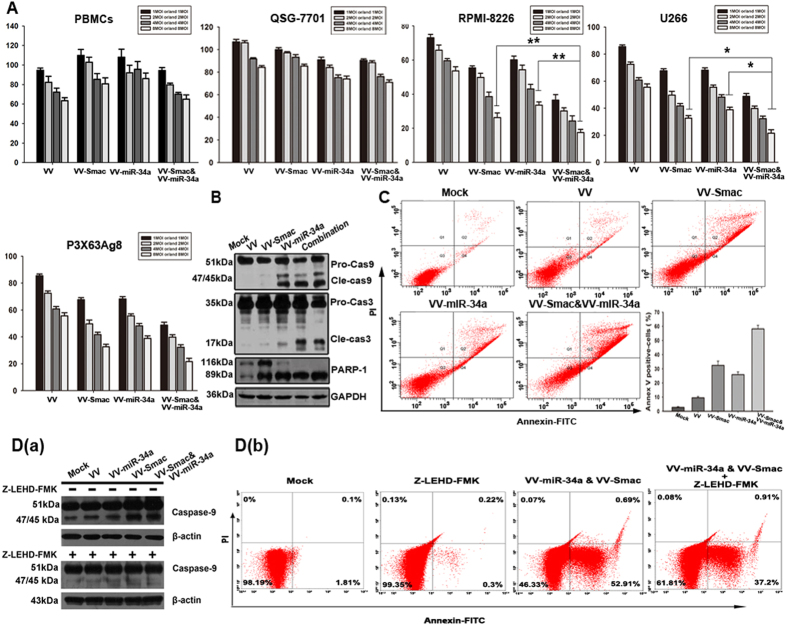
VV-miR-34a and VV-Smac synergistically induce apoptosis through activation of the caspase pathway in MM cells. (**A**) Three MM cell lines, QSG-7701 and human PBMCs were infected with VV, VV-miR-34a, VV-Smac and VV-miR-34a combined with VV-Smac at the MOI of 1, 2, 4, and 8. 72 hours later, cell viability rate was measured by MTT assay. The results were presented as the mean ± SD (n = 6) of three independent experiments. *represents *P* < 0.05, ** represents *P* < 0.005. (**B**) RPMI-8226 cells were treated with the indicated OVVs at 4 MOI. After 48 hours, whole-cell lysates were subjected to western blotting to assess the cleavage of caspase-9, -3 and PARP. (**C**) Annexin V/PI-staining method was used to detect the apoptosis induced by the indicated viruses at the MOI of 4 for 48 hours. (**D**) RPMI-8226 cells were pretreated with Z-LEHD-FMK (40uM) for 4 hours, and then infected with the indicated viruses for 48 hours at 4 MOI. Whole-cell lysates were analyzed for inhibition of activated caspase-9 by western blot (a) and apoptotic proportion by flow cytometry (b). Full length blots were shown in Fig. S1.

The effect of miR-34a and Smac expression on MM cell viability and proliferation was examined. MM cells were treated with VV-miR-34a alone, VV-Smac alone or both at the indicated doses. Cell death and proliferation was examined at 72 hours later using MTT assay. Compared with VV-miR-34a or VV-Smac treatment alone, proliferation was significantly decreased following co-infection. Taking the RPMI-8226 for example, cells survival rate kept in 26.3% at the highest dose (8 MOI) in the treatment of VV-Smac, but dramatically dropped to 17.5% when exposed to VV-miR34a at the highest dose of 8 MOI. Other two MM cell lines U266 and P3X63Ag8 presented the similar results (Fig. 2A). All of the combination indexes (CI) were less than 1 (data not show). We also infected human peripheral blood mononuclear cells (PBMCs) from healthy donors and a normal liver cell line QSG-7701 with the same doses of the indicated OVVs to verify their safety on the normal cells. Results showed that minimal cytotoxic effects were detected in normal PBMCs and normal liver cell line QSG-7701. These findings suggest that the combined forced expression of Smac and miR-34a has a specific, synergistic suppressive effect on MM cell proliferation and survival.

To determine whether the forced expression of miR-34a and Smac increase cell death by activating Smac-induced apoptosis, we examined activation of proteins in the Smac-induced caspase pathway at 48 hours after co-infection with VV-miR-34a and VV-Smac at a MOI of 4. Immunoblot analysis revealed that forced expression of miR-34a and Smac activated the cleavage of caspase-3, caspase-9 and PARP-1 (Fig. 2B). Apoptosis rates following co-infection, as determined by Annexin V/PI double staining, increased to 58.1%, nearly two times more than that of VV-Smac (32.4%) or VV-miR-34a (25.9%) treatment alone (Fig. 2C). Blocking activation of Caspase-9 by Caspase-9 inhibitor Z-LEHD-FMK (BioVison, 40 uM) inhibited apoptosis induction by co-infection with VV-miR-34a and VV-Smac. The proportion of Annexin V positive cell decreased from 52.91% to 37.2% (Fig. 2D). Taken together, the results indicate that co-expression of miR-34a and Smac induce apoptosis by activating the Smac-induced caspase pathway.

### VV-miR-34a and VV-Smac regulate expression of pro-survival proteins and protein inhibitors of apoptosis

**Figure 3:**
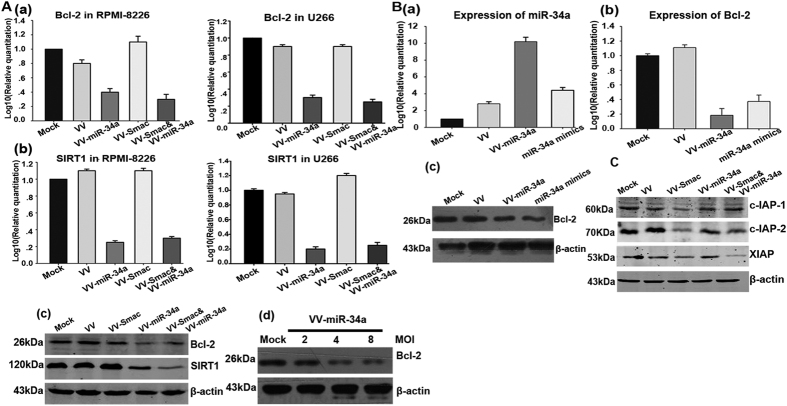
VV-miR-34a and VV-Smac regulate expression of pro-survival proteins and protein inhibitors of apoptosis. (**A**) Expression of target genes Bcl-2 and SIRT1 of miR-34a after infected with different OVVs were evaluated on mRNA and protein level. RPMI-8226 and U266 cells were treated with the indicated viruses of 4 MOI. mRNAs of Bcl-2 and SIRT1 were extracted for RT-PCR analysis (a,b). Proteins from RPMI-8226 were harvested for Western Blot analysis using the antibodies for Bcl-2 and SIRT1(c). RPMI-8226 cells were infected by VV-miR-34a with dose-dependent manner and examined the expression of Bcl-2 (d). (**B**) Increased expression of miR-34a and reduction of Bcl-2 were detected on RNA levels (a,b) and protein level (c) after RPMI-8226 infected with VV-miR-34a or transfected with mimics for 24 hours. (**C**) Immunoblot assay evaluated the inhibition of IAPs anti-apoptosis activity by VV-Smac using antibodies for c-IAP1, c-IAP2 and XIAP. Full length blots were shown in Fig. S1.

To determine the effects of miR-34a and Smac on the expression of pro-survival proteins, we also examined the expressions of miR-34a anti-apoptotic gene targets after infection. The results indicated that treatment with VV-miR-34a alone or in combination with VV-Smac both down regulated Bcl-2 and SIRT1 on an mRNA and protein level in U266 and RPMI-8226 cell lines. Moreover, miR-34a overexpression down-regulated Bcl-2 in a dose-dependent manner (Fig. 3A). A mimics of miR-34a which is a synthetic miR-34a delivered by stable nucleic acid lipid particles was used as control to further confirm that up-regulation of miR-34a resulted in a down-regulation of Bcl-2 by VV-miR-34a. Results in Fig. 3B showed that more than two times of miR-34a and Bcl-2 expression were altered in the treatment of VV-miR-34a compared with synthetic mimics, which suggests OVV is an ideal expression vector for miR-34a delivery in MM cell lines.

To further investigate the underlying mechanism of the viruses, we examined the changes in the expression of IAP family proteins (inhibitors of apoptosis proteins) after infection with the indicated viruses. Treatment with VV-Smac alone or in combination with VV-miR-34a resulted in an apparent downregulation of c-IAP1, c-IAP2 and XIAP (Fig. 3C), which demonstrated that VV-Smac inhibited the IAPs anti-apoptosis activity.

### VV-miR-34a and VV-Smac induce mitochondria-initiated apoptosis through release of cytochrome c

**Figure 4:**
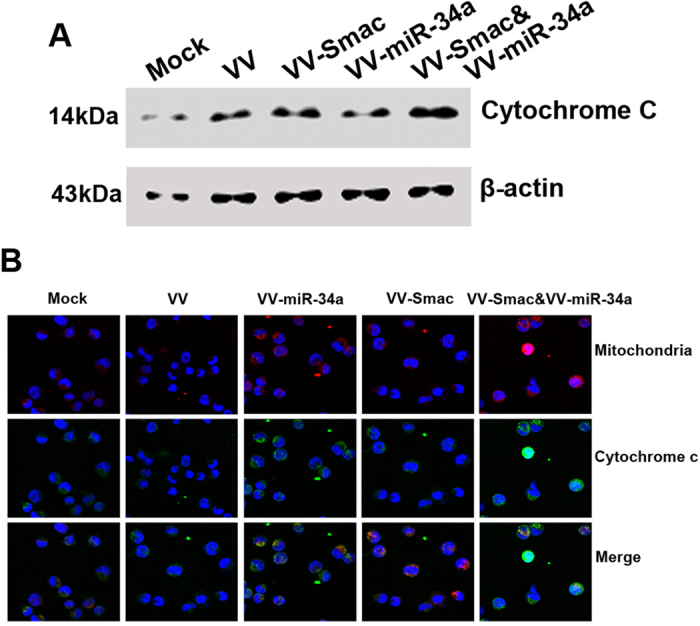
VV-miR-34a and VV-Smac induce mitochondria-initiated apoptosis through release of cytochrome c. (**A**) Up-regulation of cytochrome c was examined after infected with VV-Smac or combined with VV-miR-34a for 48 hours at 4 MOI by Western Blot. Full length blots were shown in Fig. S1. (**B**) Cell confocal microscopic images of cytochrome c (green) and mitochondria (red) stained by MitoTracker were collected from RPMI-8226 cells under treatment of VV-miR-34a, VV-Smac or the combined. Merged images showed the co-localization of cytochrome c and mitochondria (yellow).

Previous studies have reported that suppressing Bcl-2 expression increases release of cytochrome c into the cytoplasm and stimulates apoptosis correspondingly^34^,^35^. To determine whether miR-34a promotes Smac-induced apoptosis by increasing release of cytochrome c into cytoplasm, we examined changes in cytochrome c whole cell protein levels after infection with VV-miR-34a, VV-Smac or in combination, respectively. Immunoblot analysis showed that VV-miR-34a in combination with VV-Smac and VV-Smac alone stimulated upregulation of cytochrome c. However, infection with VV-miR-34a alone had no effect on cytochrome c whole cell expression (Fig. 4A). It suggests that miR-34a has not the ability to promote the whole protein upexpression of cytochrome c. To determine whether VV-miR-34a stimulates cytochrome c release from the mitochondrial endomembrane, we examined cytochrome c diffusion using laser scanning confocal microscopy (LSCM). We found that VV-miR-34a and VV-Smac infection alone can increase diffusion when compared to untreated or control VV-treated cells, while the combination synergistically increased the diffusion (Fig. 4B). These results suggest that miR-34a promotes Smac regulated apoptosis by increasing the diffusion of cytochrome c from the mitochondrial endomembrane to cytoplasm.

**Figure 5:**
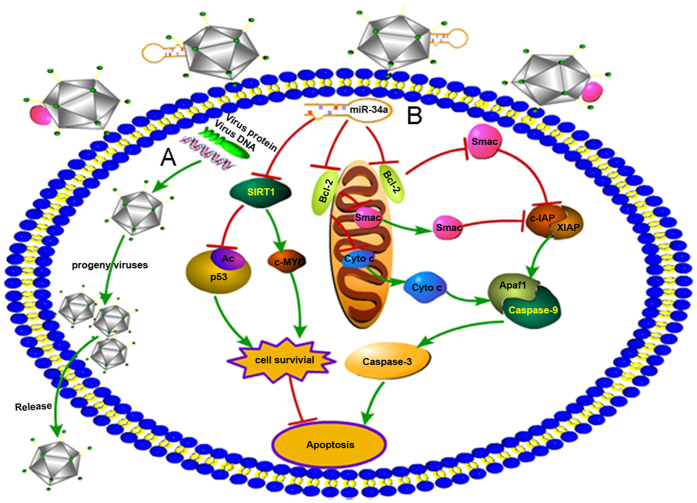
The mechanism schematic mode of viral oncolysis mediated by VV-miR-34a and VV-Smac is shown. (**A**) OVVs infect the tumor cells and elicit the tumor specific oncolytic effect. (**B**) miR-34a/Smac mediated caspase-9-dependent apoptosis pathway. Enforced expression of miR-34a by OVV blocks the function of Bcl-2, which promotes the release of cytochrome c from mitochondrial endomembrane. On the other hand, overexpression of Smac by OVV inhibits the function of inhibitors of apoptosis proteins (c-IAP, XIAP). Both of them synergistically activate the caspase-9 induced apoptosis pathway. This signal pathway image was drawn by Wen Lei.

In conclusion, our findings demonstrated that during OVVs infecting the tumor cells and eliciting the tumor specific oncolysis, enforced expression of miR-34a and Smac by VV-miR-34a and VV-Samc induced significant apoptosis in MM cells through blocking function of Bcl-2, and thus activating the release of cytochrome c from the mitochondrial endomembrane and finally synergistically enhancing the Smac-induced cytochrome c/Apaf-1/Caspase-9 signaling cascade (Fig. 5).

### Combined treatment with VV-miR-34a and VV-Smac inhibits tumor growth of MM xenograft

**Figure 6:**
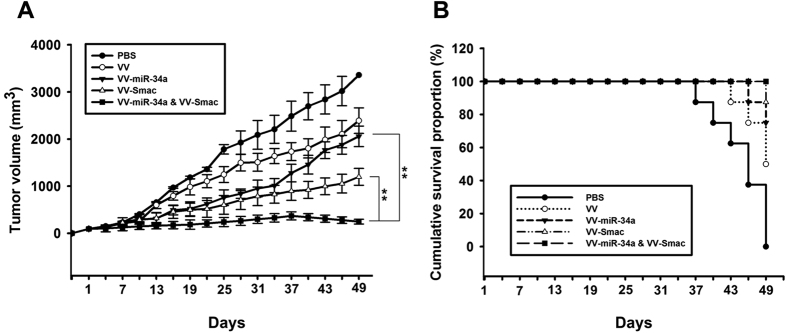
Combined treatment with VV-miR-34a and VV-Smac inhibits tumor growth of MM xenograft. (**A**) Tumor volume of various treatment groups was measured. Data are presented as means ± SD (n = 6). **P = 0.002. (**B**) Survival rate of mice was shown by the Kaplan–Meier survival curves.

To examine the therapeutic effects of treatment with VV-miR-34a and VV-Smac *in vivo*, animal experiments were performed using a MM tumor xenograft model established by RPMI-8226 cells. Tumors were measured up to postimplantation day 49 to monitor the effects of the viruses. As shown in Fig. 6A, there was a rapid decrease in mean tumor volume for animals that received intratumoral injections of VV-miR-34a, VV-Smac, or both compared to those that were injected with PBS, or the VV vector alone. The average tumor volume of PBS or VV treatment reached to 3359 mm^3^ and 2391 mm^3^, respectively, on the day before the end of the experiment. In contrast, xenografts of mice tumor volume that received VV-miR-34a, VV-Smac or the combination just reached to 2058 mm^3^, 1198 mm^3^ and 247 mm^3^, respectively. The combination treatment was more effective than VV-miR-34a (P = 0.002) and VV-Smac alone (P = 0.002). Moreover, intratumoral injection of VV-miR-34a, VV-Smac or their combination resulted in an improved survival rate compared with PBS, or VV groups (Fig. 6B). 100% of survival rate was received in the treatment of VV-miR-34a combined with VV-Smac. 75% of survival rate was obtained in the VV-miR-34a-treated mice and 87.5% of mice were still survived in VV-Smac-treatment. In comparison, only 50% of VV-treated mice were alive in the same period.

**Figure 7:**
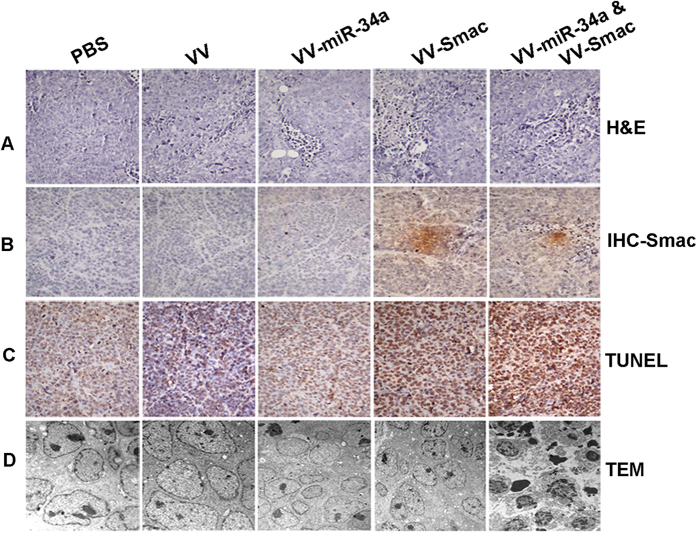
Histopathology analysis of tumor section in VV, VV-miR-34a, VV-Smac and the combined groups for RIMI-8226 xenograft tumor is shown. (**A**) Hematoxylin and eosin (HE) staining analysis. Original magnification: ×200. (**B**) IHC analysis for Smac expression in tumor tissues. Original magnification: ×200. (**C**) TUNEL assay for apoptotic cells treated with different OVVs. Original magnification: ×200. (**D**) Morphological observation of tumor tissues by TEM analysis. Original magnification: ×15000.

To evaluate the histopathology changes in the tumor tissues, Hematoxylin and eosin (H&E) and immunohistochemical (IHC) staining were performed on day 7 after viral injection. H&E staining demonstrated that the combined treatment of VV-miR-34a and VV-Smac caused more severe cytopathic effects including cell death, tumor necrosis and vessel growth inhibition, than individual treatment of them (Fig. 7A). IHC analysis utilizing anti-Smac antibody confirmed a strong expression of Smac in the tumor tissues followed treatment of combined or VV-Smac alone which verified successfully enhanced expression of Smac by OVVs in tumor mass (Fig. 7B). Apoptosis was further examined using a TUNEL assay. Apoptosis was significantly higher in tumors that received co-infection with the OVVs compared with VV-miR-34a or VV-Smac infection alone. No significant apoptosis was observed in VV or PBS treated tumors (Fig. 7C). Morphological changes in the tumors examined by Transmission electron microscope (TEM) analysis revealed that tumors injected with both VV-miR-34a and VV-Smac displayed classical characteristics of apoptosis including the appearance of apoptotic bodies, nuclear collapse, disintegration of the nuclear envelope, a greater nuclear-to-cytoplasm ratio, nucleus deformation, heterochromatin and chromatin condensation against the nuclear envelope (Fig. 7D). These results demonstrate that treatment with VV-miR-34a and VV-Smac has an inhibitory effect of tumor growth and prolongs the survival time of the mice.

## Discussion

Despite great advancements in our understanding of MM, there has been little success in treatment of the disease^36^,^37^,^38^. These tumors are resilient due in part to their genetic complexity. Therefore, targeting multiple pathways is essential for treatment of MM. In our study, we combined forced expression of miR-34a and Smac via OVV to treat MM aiming to amplify the antiproliferation effect synergistically by each other. Indeed, our study showed that the combined forced expression of miR-34a and Smac via OVV synergistically suppressed MM cell survival while sparing normal cells/tissues. Moreover, our results further verified that even though different target agents or pathways were involved in the treatment of combined strategy, synergic anti-tumor effect was still exerted as in predict, which suggests combination therapy targeting miR-34a and Smac provides an attractive strategy for treatment of MM.

Recent findings highlight miRNA therapeutics as an attractive option for treatment of MM due to their tumor suppressive activity^39^,^40^,^41^. miR-34a, a member of the miR-34 family, is one of the first miRNAs to display tumor suppressive activity. It was down-regulated in several cancer types including chronic lymphocytic leukemia^42^, colorectal cancer^43^, lung cancer^44^ and MM^19^. Moreover, recent researches have focused on its functions in modulating immune response as a putative binder of PD-L1-3′ UTR^45^. Downregulation of miR-34a contributes to several important oncogenic programs including tumor metastasis, cell apoptosis, cellular senescence as well as cell-cycle arrest in human leukemia cell lines, which suggests that miR-34a may be a novel target for treatment of human leukemia.

Here, we explored the molecular effects induced by miR-34a expression on MM cell lines via OVV. Forced expression of miR-34a increased cell apoptosis *in vitro* and *in vivo.* And one of the underlying mechanisms is to block Bcl-2, and then release cytochrome c from mitochondria to cytoplasm which stimulates the activation of caspase pathway.

Although many advantages sparked the application of miR-34a from pre-clinical research to clinical therapy, the unstable nature of its miRNA molecular structure, their rapid plasma clearance and their poor intracellular uptake have limited the use of miR-34a and other miRNAs in the clinic. Therefore, it is vital that an ideal delivery system is identified.

Our study is the first to use the novel OVV as the delivery vector for miR-34a. OVV has been an attractive agent for cancer therapy due to its ability to accept large fragments of DNA, high infectious efficiency, safety and tumor affinity, and thus overcomes the major limitations of other vehicles. Various anti-tumor genes, such as p53 or GM-CSF, are engineered into OVV vector and thus exhibit profound anti-tumor effects in several cancer cells. Up to now, several OVVs armed with anti-tumor genes are in clinical trials (Table 1). Our study showed that VV-GFP infecting MM cell line exerted significant infection efficiency. Compared with transfection of synthetic mimics, both overexpression of miR-34a and downregulation of Bcl-2 by VV-miR-34a infection were significantly higher than that of synthetic mimics, which supports OVV as an ideal delivery system for miR-34a replacement in MM.

Smac, as a novel tumor suppressor, exerts anti-tumor activity via the cytochrome c/Apaf-1/caspase-9 pathway. As MM is a genetically diverse malignance, identifying points of crosstalk between different anti-tumor gene regulated pathways is imperative. During the mitochondria-initiated caspase activation pathway, cytochrome c released from mitochondrial is critical. Its release is often blocked by overexpression of Bcl-2 in cancer cells. Here, we showed that increased release of cytochrome c and apoptosis by forced expression of Smac was enhanced by simultaneously forced expression of miR-34a. Co-treatment with VV-miR-34a and VV-Smac also synergistically suppressed proliferation and promoted apoptosis in MM cells *in vitro* and *in vivo*. Because miR-34a expression suppressed expression of Bcl-2 and enhanced release of cytochrome c from the mitochondrial endomembrane, miR-34a may significantly amplify the anti-tumor effect of Smac by enhancing Smac-induced activation of the Caspase pathway. It seems very important for target cancer therapy. Therefore, it is not surprising to find that VV-miR-34a has the ability to assist the release of cytochrome c in combination of VV-Smac and VV-miR-34a and synergistically increases the antiproliferation of VV-Smac on MM cell line *in vitro* or *in vivo*, which may provide a promising strategy for treatment of MM.

In conclusion, we successfully constructed two novel OVVs: VV-miR-34a and VV-Smac. Data from *in vitro* experiments and *in vivo* xenograft model both demonstrated that the combination of VV-miR-34a and VV-Smac exerts powerful anti-tumor activity, apoptotic effects and gene regulation. All of these findings may lay the groundwork for future application of OVV in a clinical setting.

## Materials and Methods

### Cell lines and OVVs

Human MM cell lines RPMI-8226, U266 and mouse MM cell line P3X63Ag8 were purchased from the American Type Culture Collection (ATCC, USA). Other human hematologic tumor cell lines were preserved in our lab. Human peripheral blood mononuclear cells (PBMCs) were isolated from buffy coats of healthy donors (Blood Bank, Hangzhou, Zhejiang, China) by Ficoll-Hypaque density gradient centrifugation after informed consent was signed. The study was approved by the Ethics Committee of Zhejiang University and all methods were carried out according to the approved guideline. All of the cells were cultured in RPMI-1640 culture medium supplemented with heat-inactivated fetal bovine serum (FBS, GibcoBRL, USA) at 37 °C in a humidified air atmosphere with 5% CO_2_.

OVVs used in this study include VV vector, VV-miR-34a, VV-Smac and VV-GFP. Synthetic miR-34a mimics were produced in RiboBio co., Ltd (Guangzhou, China).

### Construction, identification, purification of oncolytic vaccinia viruses

VV-miR-34a or VV-Smac was generated by homologous recombination of pCB-miR-34a or pCB-Smac with wild type VV in HEK293 cells. In details, the complete cDNA sequence of miR-34a or Smac gene was amplified by PCR (5′-GGAAGATCT CCTCCTGCATCC-3′[forward] and 5′-CCGCTTAAGATACCGCTCGAG-3′[reverse] for miR-34a, 5′-GGAAGATCTATGGCGGCTCTGAGA-3′[forward] and 5′- CCGCTTAAGTCAATCCTCACGCA -3′[reserve] for Smac). The synthetic DNA was digested with *BglII* (Takara, Japan) and *ECORI* (Takara, Japan) and then ligated into plasmid pCB to generate pCB-miR-34a or pCB-Smac, respectively. After sequence confirmation, pCB-miR-34a or pCB-Smac homologously recombined with wild type VV in HEK293 cells using Lipofectamine 2000 (Invitrogen, USA). After observing apparent cytopathic effect, cell culture medium was collected and repeated freezing and throwing three times. Obtained supernatant was VV-miR-34a or VV-Smac. Identification of correct VV was processed by plaque formation assay. Mycophenolic acid, dioxopurine and hypoxanthine were used to get rid of wild-type VV. Recombinant OVVs were amplified in HEK293 cells and purified by sucrose gradient ultracentrifugation. Moreover, viral titers were determined by TCID50 (median tissue culture infective dose).

### MTT assay

For cell proliferation assays using 3-(4,5-dimethylthiazol-2-yl) -2,5-diphenyltetrazolium bromide(MTT, Sigma, USA), cells were seeded in 96-well plates at a density of 1 × 10^5^/ml in the presence or absence of VV, VV-miR-34a, VV-Smac or VV-miR-34a combined VV-Smac in the indicated dose. After incubation for 72 hours, 20 μl of MTT solution (5 mg/ml) was added to each well and then the plates were incubated for an additional 4 hours at 37 °C. After supernatant was removed, 200 μl dimethylsulfoxide (DMSO) was added. A scientific microplate reader (Thermo, USA) was applied to detect the absorbance at a wavelength of 570 nm. Each assay was performed three times. The data was processed using Sigmaplot 6.1 package.

### Quantitative real-time amplification of RNAs

Total RNA from MM cells treated with the indicated viruses was prepared with the TRIZOL Reagent (Invitrogen) according to manufacturer’s instructions. Oligo-dT-primed cDNA was obtained using the High Capacity cDNA Reverse Transcription Kit (TOYOBO, Japan). SYBR Premix Ex Taq (TAKARA, Japan) was used as fluorescent dye. The sequence detection system (Applied Biosystems 7500, USA) was used to detect and quantify mature miR-34a and target mRNAs according to the manufacturer’s instructions. miR-34a and mRNAs were normalized on U6 and glyceraldehyde- 3-phosphate dehydrogenase (GAPDH), respectively. Comparative RT-PCR was conducted in triplicate, including no-template controls. Relative expression was calculated using the comparative cross-threshold (Ct) method. The images were obtained using the Sigmaplot 6.1 package. The primers for miR-34a and U6 were synthesized in RiboBio co., Ltd. Other primers used in this study were followed as: 5′-CTG AAG AGT TGG CTG TC-3′ (forward) and 5′-CTA AGG GAA TGA GGC-3′ (reverse) for Smac, 5′-TGATGAACCGCTTGCTAT-3′ (forward) and 5′-TGGTCTTACTTTGAGGGA-3′ (reverse) for SIRT1. 5′-CTGGTGGACAACATCGC (forward) and 5′- GGAGAAATCAAACAGAGGC-3′ (reverse) for Bcl-2, 5′-ATG GGG AAG GTG AAG GTC G-3′ (forward) and 5′-GGG TCA TTG ATG GCA ACA ATA TC-3′ (reverse) for GAPDH.

### Western blot analysis

RPMI-8226 cells were cultivated and treated with a MOI of 4 of VV, VV-miR-34a, VV-Smac or VV-miR-34a combined with VV-Smac, respectively, for 48 hours. Cells were washed twice with PBS and lysed in RIPA buffer (Sigma). Protein concentration of samples was measured by bicinchoninic acid (BCA) method. The protein samples were separated by SDS-polyacrylamide gel electrophoresis and then electroblotted onto PVDF membranes. The membranes were blocked in 5% non-fat milk for 2 hours and incubated with primary antibodies at 4 °C overnight. The membranes were washed and incubated with secondary antibody conjugated with horseradish peroxidase (1:5,000, CellSignaling Technology, USA). ECL detecting kit (Thermo Pierce, USA) was applied to visualize results. The images were obtained by Bio-Rad ChemiDox XRS imaging system. The primary antibodies and dilutions used were anti-caspase-3, anti-caspase-9, anti-PARP, anti-SIRT1, anti-Bcl-2, anti-XIAP, β-actin (1:1000, CST, USA), anti-GAPDH (1:1,000, Santa Cruz, USA), anti-cytochrome c (1:500, Abcam, UK).

### Flow cytometry analysis

RPMI-8226 cells were cultured at a density of 1 × 10^5^ cells/ml in a 6-well plate and treated with the indicated viruses at 4 MOI for 48 hours. After incubation at 37 °C, the cells were washed, resuspended in 500 μl of binding buffer and stained with 5 μl of Annexin V-FITC and 10 μl of propidiumiodide (PI) (Biouniquer, Suzhou, China) for 15 min in the dark. Then cells were examined by flow cytometry (Accuri C6, BD Biosciences, USA). The experimental data was analyzed using software of Flowjo.

### Confocal laser scanning microscopy study for cytochrome c staining

Before immunoflorescent staining for cytochrome c, MitoTracker® Red CMXRos (Invitrogen, M7512) was chosen to staining mitochondria membrane firstly. In details, RPMI-8226 cells were infected with different viruses at an MOI of 4. After 48 hours, collecting cells and stained with prewarmed (37 °C) MitoTracker® probe (100–500 nM) for 15–45 minutes. Immunoflorescent staining for cytochrome c was performed as described previously^46^. In details, cells were washed with PBS, and then fixed with 4% paraformaldehyde. After permeablized by 0.3% Triton X-100 and incubated with goat serum, cells were stained with anti-cytochrome c antibody (Abcam, 1:200 dilution) overnight at 4 °C. Then, cells were incubated with a goat anti-rabbit antibody as secondary antibody (1:500) at 37 °C for 1 h and 1 μM DAPI (SouthernBiotech, USA) for 10 min. Finally, samples were examined with a Nikon confocal microscope (Nikon C1-Si, Japan), and images were processed using NIS-Elements software package.

### Animal experiments

All animal experiments were approved by the Institutional Animal Care and Use Committee, Zhejiang University and all procedures were in according to the Guide for the Care and Use of Laboratory Animals (National Academies Press, Washington, D.C.). Female BALB/c nude mice (4-week old) were purchased from Shanghai Experimental Animal Center (Shanghai, China). For establishment of xenograft, RPMI-8226, human multiple myeloma cell lines were subcutaneously injected into the right flank of each mouse at a dose of 5 × 10^6^ cells in 100 μl RPMI-1640. When tumors reached 100–150 mm^3^ in size, mice were divided randomly into five groups (six mice per group). VV, VV-Smac, VV-miR-34a and VV-Smac combined with VV-miR-34a (1 × 10^9^ PFUs per mouse, intratumor) were injected every other day for four days. PBS treatment worked as control.

### Immunohistochemistry

Tumors were excised and fixed in 4% paraformaldehyde, embedded in paraffin, and cut in 4-mm sections. For histopathology analysis, the paraffin sections of tumors were stained with hematoxylin and eosin (H&E). For Immunohistochemical analysis, the sections were stained with rabbit monoclonal anti-Smac antibodies at 1:500 dilutions, and then washed with PBS, incubated with the avidin-biotin-peroxidase complex reagent (Vector Laboratories, USA). The slides were detected with diaminobenzidine tetrahydrochloride solution containing 0.006% hydrogen peroxide. Hematoxylin was used as a counterstain. Tissue sections stained without primary antibodies were used as negative controls. Images were acquired using a Nikon E300 inverted fluorescence microscope equipped with a Nikon digital camera (20x).

### TdT-mediated dUTP-biotin nick end-labeling (TUNEL) assay

The TUNEL method was used for detection of apoptotic cells. For this purpose, the *in situ* cell apoptosis detection kit (Roche, USA) was used. The staining was carried out according to the manufacturer’s procedures. Images were acquired using a Nikon E300 inverted fluorescence microscope equipped with a Nikon digital camera (20x).Tissue sections in PBS group were stained and served as positive controls. The TUNEL reaction preferentially labels DNA strand breaks generated during apoptosis, and allows discrimination of apoptosis from necrosis and primary DNA strand breaks induced by apoptotic agents.

### Transmission electron microscopy (TEM) analysis

For electron microscopy analysis, tumor samples (1 mm^3^) were fixed in a PBS mixture containing 2.5% glutaraldehyde overnight and then incubated in 1% osmium tetroxide for 1 h. Tissues were rinsed in ddH_2_O, dehydrated through a graded series of ethanol and propylene oxide and finally embedded in Epon 812 resin (Shell Chemicals, USA). After examination of semithin sections, areas were selected and subjected to ultrathin sectioning. Sections collected on 200 mesh copper grids were contrasted with lead citrate and uranyl acetate, examined and photographed with a JEOL 100CX transmission electron microscope (JEOL, Akishima, Japan).

### Statistical analysis

All data were expressed as the mean ± standard deviation and analyzed by SPSS 11.0 software (SPSS Inc., USA). Data analysis was performed using t-tests and P < 0.05 was considered statistically significant.

## Additional Information

**How to cite this article**: Lei, W. *et al*. Combined expression of miR-34a and Smac mediated by oncolytic vaccinia virus synergistically promote anti-tumor effects in Multiple Myeloma. *Sci. Rep.* **6**, 32174; doi: 10.1038/srep32174 (2016).

## Supplementary Material

Supplementary Information

## Figures and Tables

**Table 1:** Recent clinical trials involving OVV armed antitumor genes.

First author	Vector	Results
Mastrangelo^47^	Vaccinia-GM-CSF	Regression of injected lesions.
Marshall^48^	Vaccinia-CEA	No clinical response
Mukherjee^49^	Vaccinia-IL-2	No clinical response
Eder^50^	Vaccinia-PSA	Stabilization of PSA levels
Sanda^51^	Vaccinia-PSA	Stabilization of PSA levels
Conry^52^	Vaccinia-CEA	No clinical response
Tsang^53^	Vaccinia-CEA	No clinical response
Adams^54^	Vaccinia-HPV	Response in cervical cancer
Rochlitz^55^	MVA-Muc1	Responsein metastatic disease
Greiner^56^	rV-CEA TRICOM	Safe
