# Supplementary material for: Combined expression of miR-34a and Smac mediated by oncolytic vaccinia virus synergistically promote anti-tumor effects in Multiple Myeloma
Source: Sci Rep. 2016 Aug 24;6:32174. doi: 10.1038/srep32174 (PMC5001249; doi:10.1038/srep32174)

**Combined expression of miR-34a and Smac mediated by oncolytic vaccinia virus synergistically promote anti-tumor effects in Multiple Myeloma**

**Wen Lei^1^, Shibing Wang^2^, Chunmei Yang^1^, Xianbo Huang^1^, Zhenzhen Chen^1^, Wei He^1^, Jianping Shen^3^, Xinyuan Liu^4^ & Wenbin Qian^1,*^**


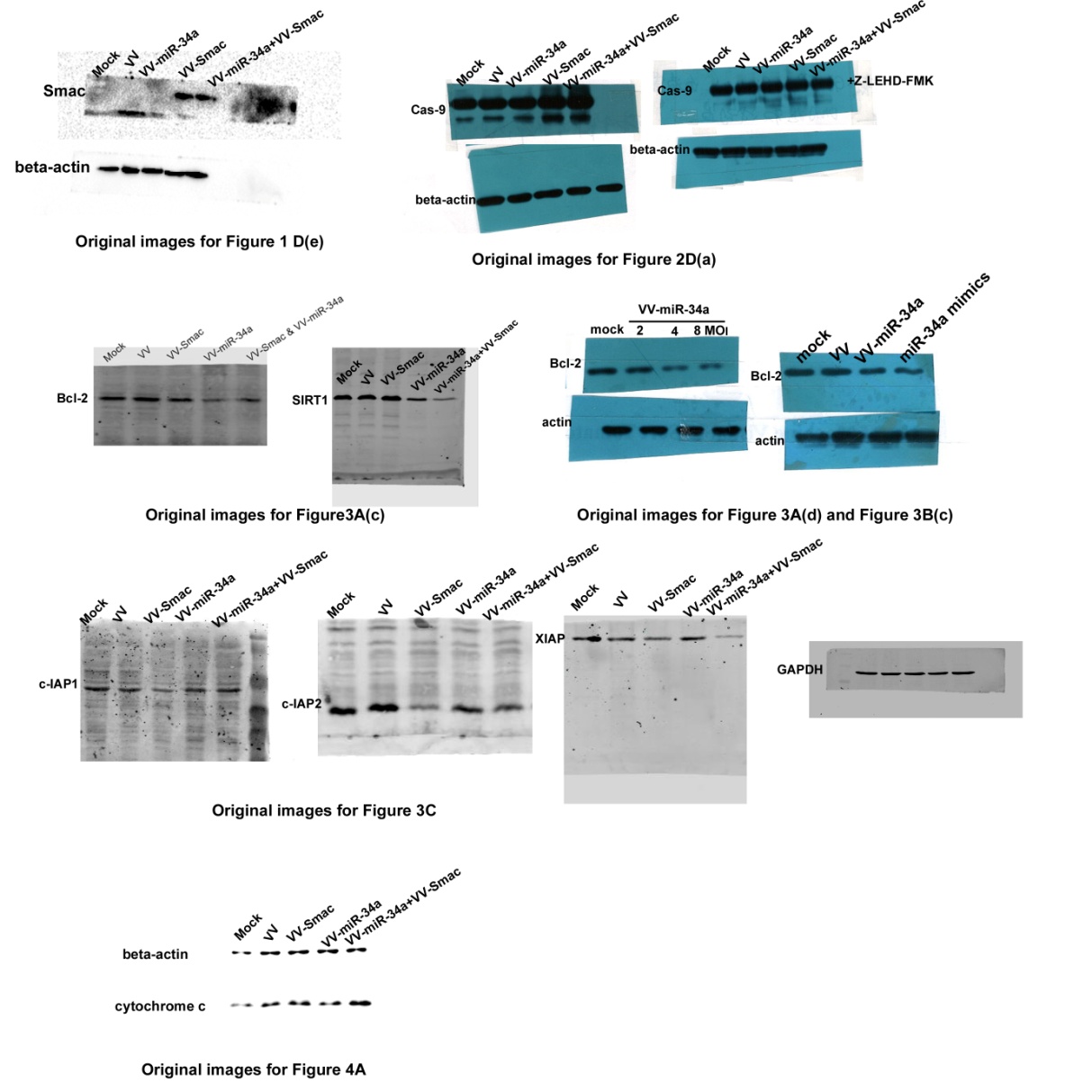

Supplement: Supplementary Information [file srep32174-s1.doc]
